# Variability analysis and inter-genotype comparison of human respiratory syncytial virus small hydrophobic gene

**DOI:** 10.1186/s12985-018-1020-9

**Published:** 2018-07-18

**Authors:** Jelena Ivancic-Jelecki, Anamarija Slovic, Sunčanica Ljubin-Sternak, Gordana Mlinarić Galinović, Dubravko Forcic

**Affiliations:** 1 Centre for Research and Knowledge Transfer in Biotechnology, University of Zagreb, Rockefellerova 10, 10000 Zagreb, Croatia; 2 Scientific Center of Excellence for Viral Immunology and Vaccines, CerVirVac, Zagreb, Croatia; 3 Teaching Institute of Public Health “Dr. Andrija Štampar”, Mirogojska 8, 10000 Zagreb, Croatia; 4 School of Medicine University of Zagreb, Šalata 3, 10000 Zagreb, Croatia; 5 Croatian National Institute of Public Health, Rockefellerova 12, 10000 Zagreb, Croatia

**Keywords:** Human respiratory syncytial virus, HVR2, Molecular epidemiology, Molecular evolution, SH, Small hydrophobic gene, Virus variability

## Abstract

**Background:**

Small hydrophobic (SH) gene is one of the mostly diverse genomic regions of human respiratory syncytial virus (HRSV). Its coding region constitutes less than 50% of the complete gene length, enabling SH gene to be highly variable and the SH protein highly conserved. In standard HRSV molecular epidemiology studies, solely sequences of the second hypervariable region of the glycoprotein gene (HVR2) are analyzed. To what extent do the strains identical in HVR2 differ elsewhere in genomes is rarely investigated. Our goal was to investigate whether diversity and inter-genotype differences observed for HVR2 are also present in the SH gene.

**Methods:**

We sequenced 198 clinical samples collected within a limited area and time frame. In this HRSV collection, rapid and significant changes in HVR2 occurred.

**Results:**

Over 20% of strains from this pool (containing HRSV genotypes NA1, ON1, GA5, BA9 and BA10) would be incorrectly assumed to be identical to another strain if only the HVR2 region was analysed. The majority of differences found in SH gene were located in the 5′ untranslated region (UTR). Seven indels were detected, one was genotype GA5 specific. An in-frame deletion of 9 nucleotides (coding for amino acids 49–51) was observed in one of group A strains. Fifteen different SH protein sequences were detected; 68% of strains possessed the consensus sequence and most of others differed from the consensus in only one amino acid (only 4 strains differed in 2 amino acids). The majority of differing amino acids in group A viruses had the same identity as the corresponding amino acids in group B strains. When analysis was restricted to strains with identical HVR2 nucleotide sequences and differing SH protein sequences, 75% of differences observed in the SH ectodomain were located within region coding for amino acids 49–51.

**Conclusions:**

Basing HRSV molecular epidemiology studies solely on HVR2 largely underestimates the complexity of circulating virus populations. In strain identification, broadening of the genomic target sequence to SH gene would provide a more comprehensive insight into viral pool versatility and its evolutionary processes.

**Electronic supplementary material:**

The online version of this article (10.1186/s12985-018-1020-9) contains supplementary material, which is available to authorized users.

## Background

Human respiratory syncytial virus (HRSV) belongs to the *Orthopneumovirus* genus of the *Pnemoviridae* family [1] and has non-segmented RNA of negative polarity as its genome. The genome is approx. 15,200 nucleotides (nts) long, comprising 10 genes that encode for 11 proteins. The HRSV strains are divided into groups A and B based on genetic and antigenic differences [2].

There are three proteins on the surface of mature HRSV virions: glycoprotein (G), fusion protein (F) and small hydrophobic (SH) protein. SH protein is present at very low amounts [3, 4]. G and F are important for attachment and fusion with the target cell, respectively [5, 6] and they can elicit the production of neutralizing antibodies [7, 8].

SH protein is a short transmembrane protein (typically 64 amino acids for group A and 65 for group B) that is anchored by a hydrophobic signal-anchor sequence near the N-terminus, with the C- terminus oriented extracellularly. The SH ectodomain is only weakly immunogenic [4, 9]. In infected cells, SH protein accumulates mostly in lipid rafts of the Golgi and of the endoplasmic reticulum [3]. It is a viroporin, forming a transmembrane pentameric ring that functions as a channel for cations and small molecules [10, 11]. HRSV SH protein blocks or delays apoptosis through inhibition of the TNF-α signalling pathway [12] but the mechanism is still not fully elucidated.

In molecular epidemiology studies, HRSV surveillance and genotyping are based on sequences of the second, C-terminal hypervariable region of the G gene (HVR2). In frame duplications of 72 and 60 nts have occurred within this region, leading to emergence of group A strains belonging to genotype ON1 (which originated from NA1, and was firstly detected in 2010 [13]) and of group B strains belonging to BA genotypes, respectively.

To what extent do the strains identical in HVR2 differ elsewhere in their genomes is rarely investigated. In the analysis by Agoti et al. such strains differed by at least 1 and up to 9 nts across the complete genome [14].

Next to HVR2, the most diverse HRSV genomic region is the SH gene [15]. Unlike all other HRSV genes, the SH coding region constitutes less than 50% of the complete gene length (the coding regions of other HRSV genes constitute ca. 75–99% of the gene length). Following our previous analysis of HVR2 sequences of strains detected within a limited geographical area (the Zagreb region) and limited time frame (March 2011 to March 2014) [16], the goal of this study was to investigate whether virus variability and inter-genotype differences observed for HVR2 are also present in the SH gene. Within this pool of viruses, rapid and significant genetic changes have occurred in HVR2 [16]. The subset of strains analysed in this research belong to 3 HRSV A (NA1, ON1 and GA5) and 2 HRSV B genotypes (BA9 and BA10) and it included all the genotypes that were detected in Zagreb, 03/2011–03/2014. These are also the most common HRSV genotypes detected worldwide in the last decade.

## Methods

### Clinical samples

Nasopharyngeal secretions were obtained from children who were in-patients with acute respiratory infections, hospitalized mostly due to bronchiolitis or pneumonia [16]. Based on the availability of the material, 220 samples with previously determined sequences of the HVR2 region and genotype [16] were chosen for SH gene analysis. Sequences were obtained from 198 samples.

### Reverse transcription, PCR and sequencing

Total RNA was extracted from 500 μL of clinical samples according to the method reported by Chomczynski and Mackey [17]. Reverse transcription was performed at 42 °C for 60 min, in a reaction mix containing 10 μL of isolated RNA, 1× PCR buffer (GE Healthcare, UK), 0.1 mM of each dNTP, 20 U of RNase inhibitor (Thermo Fisher Scientific, USA), 1.25 mM MgCl_2_, 2.5 mM of random hexanucleotide primers and 50 U of MuLV reverse transcriptase (Thermo Fisher Scientific, USA) in a final volume of 20 μL.

Nested PCR was carried out with two sets of primers. For the first amplification, forward SH1 (5′ CACAGTKACTGACAAYAAAGGAGC 3′) and reverse F164 (5′ GTTATGACACTGGTATACCAACC 3′) primers were used, followed by the second amplification with the SH3 (5′ CAGATCATCCCAAGTCATT 3′) and SH4 (5′ TGATTGAGAGTGTCCCAGGT 3′) primer pair for HRSV A strains and the SH5 (5′ AGCCATTGTCTGCCAGAYCTAGAG 3′) and SH4 primer pair for HRSV B strains.

Ten microliters of the reverse transcription reaction mix was used for the first amplification, whereas 2 μL of the first amplification mixture was used for the second PCR. PCR reaction mixtures contained 1× PCR buffer (GE Healthcare, UK), 10 μM of each dNTP, 0.25 mM MgCl_2_, 0.25 μM of each primer and 5 U of *Taq* polymerase (GE Healthcare, UK). PCR conditions for the first amplification were: 95 °C for 5 min, 40 cycles of 95 °C/30 s, 50 °C/30 s, 68 °C/2 min, followed by final extension at 68 °C for 7 min. The second PCR was performed under the same conditions, except the primer annealing temperature was 52 °C, and the extension step was shortened to 1 min.

The amplification products of ca. 600 nts were separated on a 1.5% agarose gel. Sequencing reactions were set up with gel purified DNA, one of the specific primers used in the second PCR and a BigDye Terminator v3.1 Cycle Sequencing Kit (Thermo Fisher Scientific, USA) according to the manufacturer’s protocol. Sequencing and sequence analysis were performed on a 3130 Genetic Analyzer (Thermo Fisher Scientific, USA). Obtained sequences were submitted to National Center for Biotechnology Information (NCBI) database, under acc. Nos. MF479446 - MF479565 (HRSV A) and MF479566 - MF479643 (HRSV B).

The HRSV genomic region used in the analyses (referred to as SHseg) spanned bases 4172–4642 of strain A2 (acc. no. M11486) and 4218–4689 of B1 (acc. no. AF013254), which are the prototype strains of group A and B, respectively.

### Collection of HVR2 sequences

HVR2 sequences included in this study are a subset of sequences determined during our previous study on HRSV molecular epidemiology [16]. HVR2 region spanned bases 5274–5543 of strain A2 and 652–981 of BA4128/99B. The length of HVR2 is 270 or 242 nts for group A and 270 or 330 nts for group B.

### Sequences retrieved from NCBI database

All available HRSV sequences that possessed AAAAAAGTCA at the end of SH 3′ untranslated region (UTR) and beginning of SH-G intergenic region were downloaded from NCBI database. Besides the 3 strains sequenced in this study, 14 sequences were found, acc. Nos. KF826826, KJ672447, KJ672462, KJ672474, KJ672479, KJ672483, KU950473, KU950479, KU950487, KU950501, KU950564, KU950609, KU950616 and KX765891.

### Multiple sequence alignments and analyses

Sequences were aligned using BioEdit, version 7.2.5 and ClustalX 2.1. Sequence conservation (defined as the percentage of genomic positions identical in all strains; gaps were ignored during computing) and mean *p*-distance (proportion of nucleotide sites at which two sequences being compared are different) were calculated using MEGA software v6.06.

The ratio of the number of non-synonymous substitutions per non-synonymous site and synonymous substitutions per synonymous site (dN/dS) was calculated with the single-likelihood ancestor counting method (SLAC) available on the Datamonkey server (http://www.datamonkey.org/).

### Phylogenetic analysis

Phylogenetic trees were generated using the maximum likelihood method with MEGA software v6.06, under the most appropriate model of nucleotide substitution determined with jModeltest v2.1.4. Bootstrap probabilities for 1000 iterations were calculated to evaluate confidence estimates. Values higher than 70% were considered significant.

### Selective pressure and glycosylation analysis

Codon-based analysis of selective pressure for SH gene sequences was performed using the HyPhy package available on the Datamonkey server. Four different methods were used: SLAC, fixed-effects likelihood, internal branch fixed-effects likelihood and mixed effects model of evolution. Sites were considered positively or negatively selected if (a) they met the cut-off criteria of *p*-value of < 0.1; or (b) they were recognized by at least two methods.

NetOGlyc 4.0 server (http://www.cbs.dtu.dk/services/NetOGlyc/) and NetNGlyc 1.0 servers (http://www.cbs.dtu.dk/services/NetNGlyc/) were used for O- and N-glycosylation site prediction, respectively.

## Results

### Sequence variability and phylogeny

**Fig. 1:**
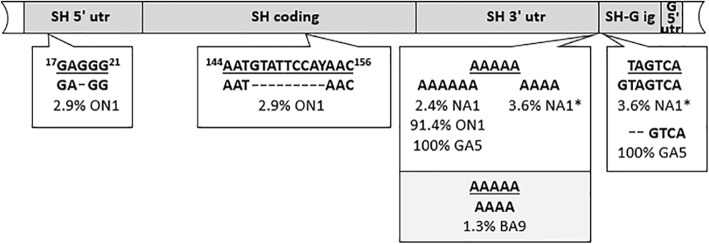
Variations in length detected in SHseg. Variations in group A strains are shown in white rectangles; in group B strains in gray. Underlined sequences are consensus sequences. The percentage of strains possessing presented non-consensus sequences is shown. Asterisks indicate that both variations were in the same NA1 strain

The SHseg spanned the entire SH gene (coding, 5’ UTR and 3’ UTR), SH-G intergenic region and G gene 5’ UTR (Fig. 1). We obtained sequences of 120 HRSV A strains; 83, 34 and 3 were NA1, ON1 and GA5 strains, respectively. Seventy-eight sequences belonged to HRSV B group; 77 belonged to genotype BA9 and 1 to BA10. The nucleotide length of the consensus sequence was 469 nts for group A and 472 nts for group B strains. Seven indels were detected (Fig. 1). In group A deletions were at positions 4190, 4318–4316, in poly A tail (4577–4581) and at positions 4582–4583 in reference to the consensus sequence A2; insertions were an additional adenosine in poly A tail and a guanosine after nucleotide 4581 (Fig. 1). In group B, a deletion of an adenosine in poly A tail was observed (position 4626–4630 in reference to the consensus sequence B1). Five indels were found only within a single genotype, but in very low percentages. Deletion of 2 nts at the beginning of the SH-G intergenic region in GA5 strains, preceded by 6 adenosines^1^ was found in 100% of our GA5 strains and only in GA5 genotype (and therefore could be classified as genotype specific). Our viral pool contained only 3 GA5 strains, identical in both HVR2 and SHseg and all collected in February 2014. Therefore we searched for other HRSV sequences that possess AAAAAAGTCA at the end of SH 3’ UTR and beginning of SH-G intergenic region in NCBI database. Fourteen sequences were retrieved and all belong to genotype GA5 (Additional file 1: Figure S1).

We analysed if viruses with identical HVR2 sequences (considered to be identical viruses in standard molecular evolutionary analyses) had differences in SHseg. Some strains identical at HVR2 differed at SHseg, and vice versa, strains different at HVR2 were identical at SHseg (Table 1, Additional file 2: Table S1). In our HRSV pool, if strains were inferred as being the same based only on HVR2 sequence analysis, 44 strains (22.3%) would have been falsely characterized.

**Table 1:** Comparison of HRSV sequence clustering based on HVR2 vs clustering based on SHseg

HRSV group	A			B	
genotype	NA1	ON1	GA5	BA9	BA10
total no. of strains	83	34	3	77	1
of different HVR2 sequences	37	22	1	31	n.a.^a^
no. of different SHseg sequences	30	20	1	21	n.a.
no. of strains identical at HVR2 that are also identical at SHseg (divided in groups of identical sequences)	45 (3 + 2 + 6 + 2 + 3 + 2 + 13 +3 + 2 + 3 + 2 + 2 + 2)	14 (2 + 2 + 2 + 3 + 3 + 2)	3 (3)	44 (5 + 8 + 8 + 2 + 2 + 15 + 2 + 2)	n.a.
no. of strains possessing unique sequences at HVR2 and at SHseg	11	11	0	7	n.a.
no. of strains identical at HVR2 and different at SHseg (divided in groups of identical HVR2 sequences)	19 (4 + 2 + 2 + 4 + 5 + 2)	8 (2 + 2 + 2 + 2)	0	17 (2 + 2 + 2 + 2 + 4 + 3 + 2)	n.a.
no. of strains identical at SHseg and different at HVR2 (divided in groups of identical SHseg sequences)	26 (7 + 9 + 4 + 2 + 2 + 2)	7 (7)	0	28 (3 + 6 + 4 + 6 + 3 + 2 + 2 + 2)	n.a.

^a^*n.a.* not applicable

As expected, none of SHseg sequences found among HRSV A strains was detected within HRSV B pool, but there was also no overlapping between the genotypes. *I. e.*, none of the SHseg sequences present in any of the 5 HRSV genotypes included in the study were detected in strains belonging to another genotype.

**Fig. 2:**
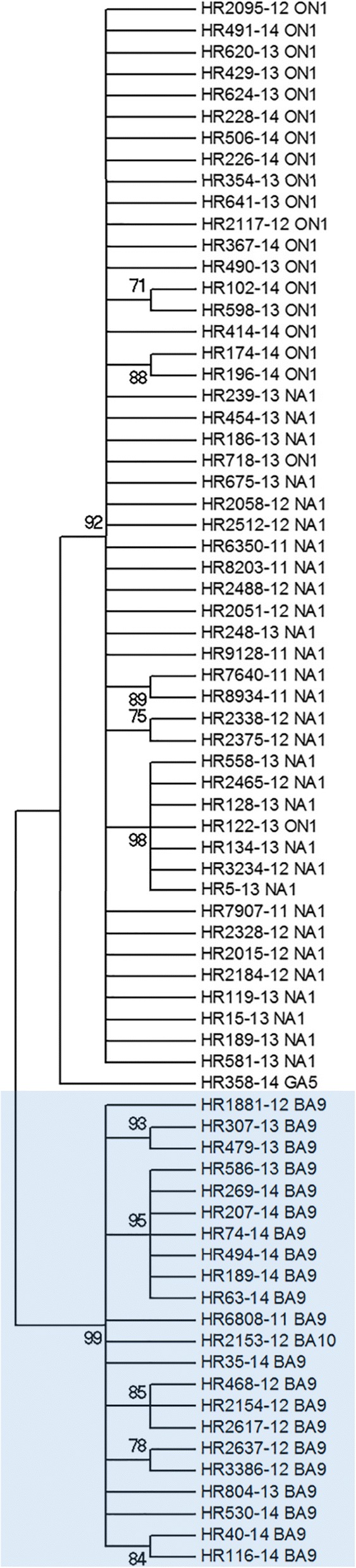
Phylogenetic analysis based on SHseg. The tree was generated using the Maximum Likelihood method, General Time Reversible model. The numbers are bootstrap values determined for 1000 iterations; the tree was condensed using a cut-off value of 70%. Strain genotypes (determined by phylogenetic analysis based on HVR2) are shown next to strain names. Blue background indicates group B strains

The phylogenetic analysis based on SHseg (Fig. 2) did not discriminate NA1 and ON1 genotypes. NA1 and ON1 strains were placed within a common clade, separated from GA5 strains and from group B lineage. In group B, a single BA10 strain is positioned among the BA9 strains (Fig. 2).

### Nucleotide variability

We calculated the percentage of conservation and mean *p*-distance within complete HVR2, complete SHseg and within each individual region of the SHseg (Table 2). Compared to HVR2, SHseg showed higher sequence conservation in all genotypes. Difference in variability was especially evident when solely the coding region of the SH gene was compared to HVR2 (HVR2 consists of 96.5% of coding region (for ON1), 95.6% or 96.7% (for NA1) and 90.9% or 97.3% (for BA9)). The evolutionarily newest genotype ON1 showed the highest conservation percentage and the lowest *p*-distance values (not taking into account 3 GA5 strains). When individual genomic regions within SHseg were compared, differences among genotypes were observed: all HRSV A strains were identical at the 5’ UTR of the G gene, but ON1 strains showed a smaller percentage of conserved sites in the 5’ UTR of the SH gene, compared to other regions. In NA1 strains, the conservation percentage was similar throughout the SHseg (differing by no more than 4.6%, excluding the 5’ UTR of the G gene). Among BA9 strains, a smaller percentage of conserved sites was again observed in the 5’ UTR of the SH gene, while the values for other regions were comparable.

**Table 2:** Conservation percentage and mean *p*-distance for HVR2, complete SHseg and individual genomic regions within SHseg

group	genotype (no. of samples)	HVR2	SHseg	individual SHseg genomic regions (max. Length in nt for HRSV A and HRSV B, respectively)				
				SH gene 5’ UTR^a^ (84, 85)	SH coding (195, 198)	SH gene 3’ UTR (132, 130)	intergenic region (45, 44)	G gene 5’ UTR (15,15)
A	ON1 (34)							
	a	88.0	91.5	81.0	94.4	90.2	90.9	100
	b	0.017	0.007	0.019	0.004	0.009	0.007	0
	NA1 (83)							
	a	78.5	89.8	88.1	91.3	88.6	86.7	100
	b	0.028	0.015	0.025	0.011	0.018	0.007	0
	overall^b^ (120)							
	a	67.3	79.4	71.4	85.1	76.5	71.1	100
	b	0.038	0.018	0.029	0.012	0.023	0.013	0
B	BA9 (77)							
	a	81.5	89.6	78.8	93.9	89.2	90.9	93.3
	b	0.023	0.020	0.040	0.011	0.021	0.015	0.032
	overall^c^ (78)							
	a	78.8	87.5	76.5	92.9	88.5	79.5	93.3
	b	0.023	0.020	0.040	0.011	0.021	0.017	0.032

^a^UTR, untranslated region

^b^group A includes strains belonging to ON1, NA1 and GA5 (3 strains identical in HVR2 and in SHseg)

^c^group B includes BA9 strains and a single BA10 strain

Unlike group A, the 5’ UTR of the group B G gene was not completely conserved, but the only difference among 78 strains was in the last nucleotide of this region; 60% of strains had a cytosine and 40% an adenine.

### Variability of protein sequences

**Fig. 3:**
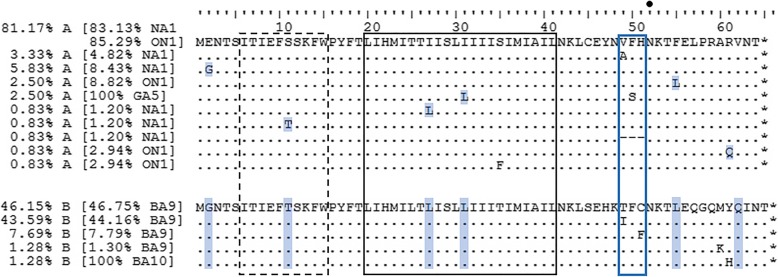
Alignment of deduced SH protein sequences. Dashed line, solid black line and blue line rectangles indicate 10 residue-long conserved fragment in the cytoplasmic domain Torres et al. [32], transmembrane region Li et al. [30] and a putative antigenic site, respectively. Asterisk shows the position of stop codons; − indicates gap. Black dot shows predicted N-glycosylation site. Blue shadings indicate amino acids that differ from the most common one in group A and have the same identity as corresponding amino acids in group B strains

The strains analysed in this study possessed 10 and 5 different SH protein sequences in the HRSV A and HRSV B strains, respectively (Fig. 3). The SH protein sequences identical to the group A or B consensuses was obtained in 67.7% of strains. The others differed from the consensuses in only one amino acid (Fig. 3), with two exceptions: a) the 3 GA5 strains differed in 2 amino acids; b) in a single strain, RSV5–13 belonging to genotype NA1, amino acids 49–51 are missing. A single N-glycosylation site was predicted for all strains (the same one, Fig. 3); no potential O-glycosylation sites were recognised.

In the intracellular and transmembrane protein region (amino acids 1–41), variability was seen only in group A strains and 4 out of 5 observed differences were such that differing amino acids had the same identity as corresponding amino acid in group B strains (shaded areas in Fig. 3). Sequence coding for the fifth amino acid difference, S35F, observed in only one ON1 strain (HR226–14 from 2014), presents so far a unique SH gene sequence, when compared to all sequences available in open public databases.

Greater versatility, including the deletion of 3 amino acids, was observed in the SH ectodomain. Again, two amino acid differences observed within HRSV A strains were such that differing amino acids had the same identity as the corresponding amino acids in group B strains (shaded areas in Fig. 3). Among the HRSV B strains, differences in the amino acid sequence were seen only within the SH ectodomain. Out of 9 amino acid differences observes in the SH ectodomain (in both groups, Fig. 3), 5 were in protein region 49–51.

The codon-based analysis of selective pressure did not identify any positively selected codon and the mean dN/dS values were low: 0.13, 0.29 and 0.15 for the NA1, ON1 and BA9 genotypes, respectively, indicating that substitutions are not favoured and are purified from the population.

The last analysis was restricted to strains identical in HVR2 nucleotide sequences and differing in SH protein sequences. Eight groups of such strains were identified (Table 3), in 4 of them the differences were in the SH ectodomain. From those 4, in 3 groups (2 HRSV A and 1 HRSV B), the differences were located within the SH protein region 49–51, including the case where these 3 amino acids were missing.

**Table 3:** Differences between SH proteins among strains identical at the HVR2 region

Strains identical in HVR2 nucleotide sequences and differing in SH protein sequences	Group	Genotype	Amino acid position	Amino acid identity	SH protein region
HR15–13	A	NA1	2	G	cytoplasmic
HR186–13				E	
HR119–13	A	NA1	2	E	cytoplasmic
HR129–13				G	
HR175–13	A	NA1	2	G	cytoplasmic
HR372–13				E	
HR116–13	A	NA1	11	S	cytoplasmic
HR134–13				T	
HR2033–12	A	NA1	49	V	ectodomain
HR110–15				A	
RSV3234–12	A	NA1	49–51	VFH	ectodomain
RSV5–13				–	
HR40–14	B	BA9	49, 51	T, F	ectodomain
HR189–14				I, C	
HR2154–12	B	BA9	60	K	ectodomain
HR2253–12				M	

Legend:- gap

## Discussion

Due to the fact that SH gene UTRs are relatively long (when compared to the coding region of the gene) and that the majority of mutations occur in them, HRSV SH gene is characterized by high variability while the SH protein remains highly conserved.

Besides the complete SH transcript, the genomic segment we analysed also included its two downstream regions, stopping immediately before the start codon of the G protein mRNA. The last two regions were included because they are known to play a role in the transcriptional regulation of both upstream and downstream genes [18–20].

We analysed whether a difference in the diversity of the SH/G junction (i.e. of the sequences between the stop codon of the upstream gene and the start codon of the downstream gene) among HRSV genotypes could be observed. A sequence characteristic only for GA5 strains was observed, but not all previously reported GA5 strains possess this sequence [20]. Whether it represents an evolutionary novelty, specific for recent GA5 strains requires further investigation (the oldest GA5 strain in our analysis was from Mexico, from 2004).

The overall nucleotide diversity of SHseg was investigated using two methods: conservation percentage that is not influenced by the number of identical sequences; and *p*-distance that is, and therefore, defines more precisely diversity within this particular HRSV population. The results of both methods were concordant. The level of genetic diversity observed in SHseg was not comparable to the one detected in HVR2, except in the 5’ UTR of the genotypes with prolonged G gene (ON1 and BA9) which gained striking dominance within the short timespan we analysed. On the contrary, the other SH UTR and SH-G intergenic region showed less variability in ON1 and BA9 strains, when compared to NA1 genotype**.**

The current HRSV genotyping and molecular surveillance system is based on HVR2 [21, 22], a quite short genomic segment. It partitions HRSV strains in over 15 group A and over 20 group B genotypes. Regarding phylogeny, SHseg in not as informative as HVR2. E.g. in analyses based on HVR2, ON1 and NA1 strains are placed on different branches, even when one copy of the duplicated segment is excluded from the analysis [23]. Still, basing molecular epidemiology studies only on HVR2 may be quite misleading. In our pool of HRSV strains, over 20% of them would be incorrectly assumed to be identical to another strain if only the HVR2 region was analysed. An alternative HRSV genotyping scheme has recently been proposed [24], based on the complete G protein ectodomainwhich is twice longer than HVR2. Although this would increase the discrimination between strains, it is still focused solely on one protein gene. The G gene is non-representative of the HRSV genome, as it is marked by elevated substitution rates (attributed to relaxed selective constraints [25]) and a flip-flop substitution pattern [26]. Broadening the target genome sequence to SH (whose length in our analysis was 401–411 nts in group A, 412 or 413 in group B) or only to SH 5’ UTR (in our analysis, this was a region of 83 or 84 nts in group A, 85 in group B) would provide better insight into the interrelation between molecular epidemiology and molecular and evolutionary dynamics.

After the translation of our genomic sequences, only 15 different protein sequences were obtained. Although group A and group B strains diverged approximately 350 years ago [27], the majority of amino acids which differ from the consensus in group A have the same identity as corresponding amino acids in group B strains. This indicates that the variability of the SH protein is not only low, but is also restricted. Based on the fact that HVR2 has the highest evolutionary rates, we concentrated on strains possessing the same HVR2 nucleotide sequences, but still differing in their SH protein sequences. Those are the strains most likely to be highly similar and therefore we hypothesized that the observed differences could be a result of selective pressure. Out of 4 sets of strains with differences in the SH ectodomain, in 3 sets the differences were in SH protein region 49–51. In fact, besides S35F the only amino acid differences observed in group A strains which differed from the group B consensus, were located within 49–51 region. In our viral pool, which was very limited both geographically and temporally, 6.7% of group A strains and 51.3% of group B strains possessed mutation in region 49–51.

Our hypothesis that SH region 49–51 is an antigenic site still needs confirmation, but the same amino acids were shown to be highly variable by Lima et al. [28]. Their analysis was performed on strains belonging to HRSV genotypes different from ours and detected in Brazil in 2004–2005. Besides finding T49I variability (as observed by us), P and A at position 49 were also reported among group B strains. Chen et al. [29] detected variability in the same region in American HRSV strains from 1998 to 1999 [29]. In group A, the most common amino acid at position 49 was V (the same as in our analysis), but I was also observed. Among group B strains, at position 51, besides the most common amino acid C and F (the same as in our analysis), Chen et al. also identified Y [29].

A deletion of 9 nts coding for amino acids 49–51 was found in the SH gene sequence of an NA1 strain from January 2013. The deletion was found only in this strain that caused a bronchiolitis in a 2-month-old child. The deletion was confirmed by 3 independent RNA isolations and preparations of DNA for sequencing. During the same HRSV epidemic, strain RSV3234–12 (from November 2012) was detected in a sample from a 4-month-old child with bronchiolitis. The two strains are identical in the SH, G an F genes, except for the fact that RSV3234–12 does not possess this deletion in the SH gene. Other genomic regions were not sequenced. In group A strains, H51 (which is missing from RSV5–13) is a crucial residue in the regulation of SH ion channel activity [30]. Together with H22, H51 plays a key role in the opening and closing mechanism of the SH pentameric pore, since they are located in strategic places within the chains, close to the N- and C-termini [30, 31]. An alternative amino acid at position 51 (Y, a non-basic amino acid) has been reported before, in a Brazilian GA5 strain from 2004 [28], but so far there were no reports of the deletion observed in RSV5–13. In vitro experiments regarding the functionality of the SH protein from RSV5–13 or modelling studies that would compare its structure to other HRSV SH proteins have not been performed.

## Conclusions

Basing HRSV molecular epidemiology studies solely on HVR2 largely underestimates the complexity of circulating virus populations. In strain identification, broadening of the genomic target sequence to SH gene would provide a more comprehensive insight into viral pool versatility.

Unlike its gene, SH protein is characterized by intra-group conservation and by highly restricted inter-group variability except for amino acids 49–51, implicating that this protein region may be relevant for the antigenicity of the virus.

## Additional files

Additional file 1: **Figure S1.** Phylogenetic tree of RSV strains based on HVR2 genomic segment. Tree was generated using maximum-likelihood method, based on the General Time Reversible model and discrete gamma distributed rates across sites. The scale bar indicates the proportion of nucleotide substitutions per site. Numbers are percentages of bootstrap values determined for 1000 iterations, only values above 70% are shown. Strain designations are composed of NCBI GenBank acc. no., name and genotype. (PDF 54 kb)

Additional file 2: **Table S1.** Sequences identical at HVR2 or SHseg. (PDF 82 kb)

## Abbreviations

dN/dS: The ratio of number of non-synonymous substitutions per non-synonymous site and synonymous substitutions per synonymous site
F: Fusion protein
G: Glycoprotein
HRSV: Human respiratory syncytial virus
HVR2: The second hypervariable region of the glycoprotein gene
NCBI: National Center for Biotechnology Information
nts: Nucleotides
SH: Small hydrophobic
SLAC: Single-likelihood ancestor counting method
UTR: Untranslated region

## Footnotes

1 All sequences are presented as plus strands, in the 5′ to 3′ orientation.
