# Supplementary figures and images for: Variability analysis and inter-genotype comparison of human respiratory syncytial virus small hydrophobic gene
Source: Virol J. 2018 Jul 18;15:109. doi: 10.1186/s12985-018-1020-9 (PMC6052705; doi:10.1186/s12985-018-1020-9)

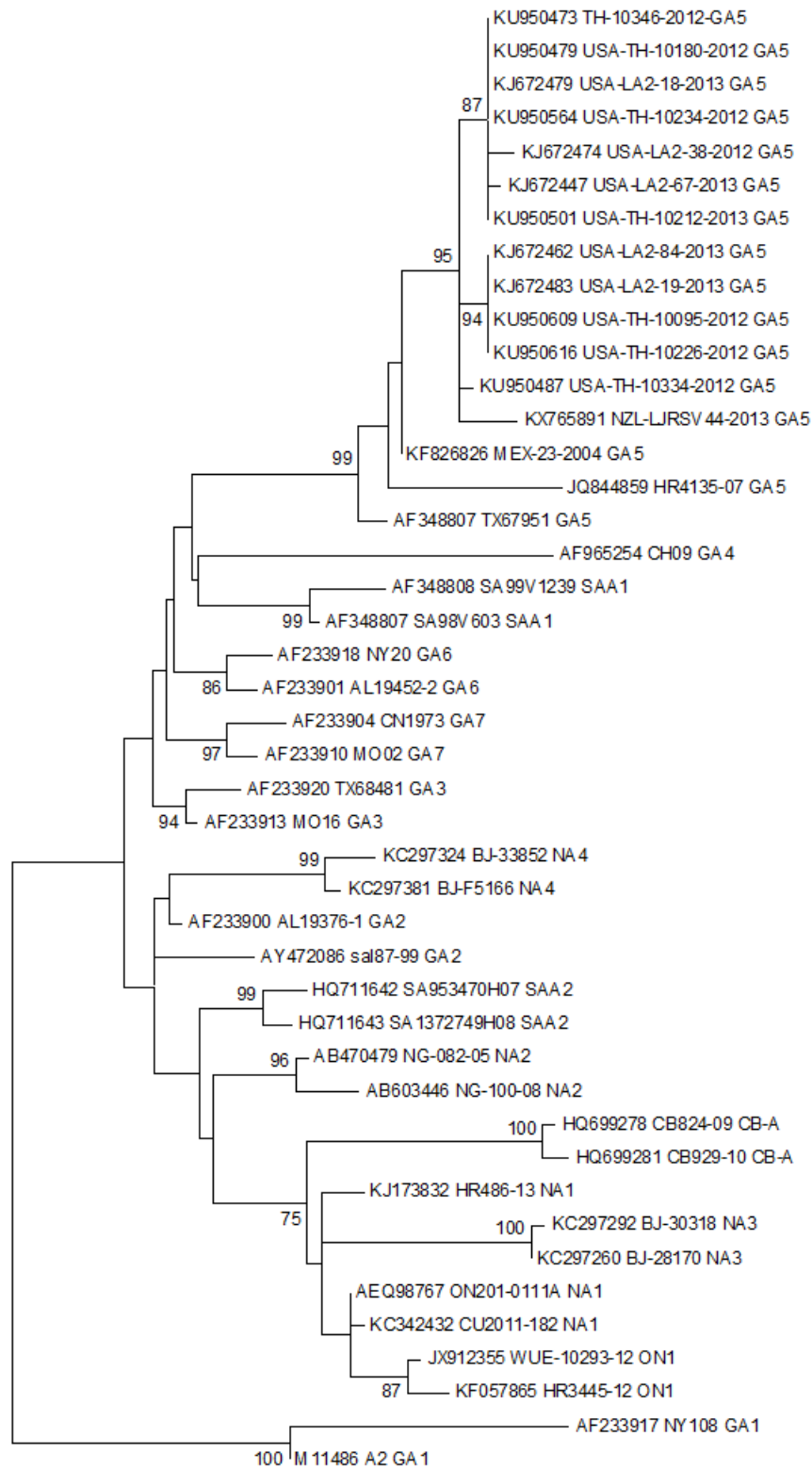

0.02

Supplement: Supplementary file 1 — Figure S1. Phylogenetic tree of RSV strains based on HVR2 genomic segment. Tree was generated using maximum-likelihood method, based on the General Time Reversible model and discrete gamma distributed rates across sites. The scale bar indicates the proportion of nucleotide substitutions per site. Numbers are percentages of bootstrap values determined for 1000 iterations, only values above 70% are shown. Strain designations are composed of NCBI GenBank acc. no., name and genotype. (PDF 54 kb) [file 12985_2018_1020_MOESM1_ESM.pdf]
