# Supplementary material for: Variability analysis and inter-genotype comparison of human respiratory syncytial virus small hydrophobic gene
Source: Virol J. 2018 Jul 18;15:109. doi: 10.1186/s12985-018-1020-9 (PMC6052705; doi:10.1186/s12985-018-1020-9)
Supplement: Supplementary file 2 — Table S1. Sequences identical at HVR2 or SHseg. (PDF 82 kb) [file 12985_2018_1020_MOESM2_ESM.pdf]

HRSV-A

sequences identical in HVR2

genotype NA1

group	strain name
1.	HR6350-11
2.	HR7640-11
3.	HR7907-11
4.	HR8203-11
5.	HR8934-11
6.	HR9128-11
7.	HR16630-11
8.	HR19632-11
	HR2083-12
	HR2100-12
9.	HR2015-12
	HR2176-12
10.	HR2033-12
	HR2034-12
	HR2089-12
	HR2125-12
	HR285-12
	HR2202-12
	HR2488-12
	HR2512-12
	HR110-15
	HR239-13
11.	HR2035-12
12.	HR2051-12
13.	HR2058-12
	HR2191-12
	HR2249-12
14.	HR2146-12
15.	HR2184-12
16.	HR2199-12
	HR2308-12
17.	HR2287-12
18.	HR2328-12
19.	HR2338-12
20.	HR2375-12
21.	HR2433-12
22.	HR2465-12
23.	HR2666-12
24.	HR3234-12
	HR5-13
25.	HR15-13
	HR186-13
26.	HR41-13
27.	HR100-13
	HR116-13

sequences identical in SHseg

group	strain name
1.	HR100-13
	HR116-13
	HR130-13
	HR135-13
	HR146-13
	HR162-13
	HR171-13
	HR172-13
	HR206-13
	HR250-13
	HR280-13
	HR518-13
	HR552-13
	HR555-13
	HR556-13
	HR590-13
	HR593-13
	HR623-13
	HR633-13
	HR655-13
	HR3234-12
2.	HR6350-11
	HR16630-11
	HR19632-11
	HR2033-12
	HR2034-12
	HR2035-12
	HR2083-12
	HR2089-12
	HR2100-12
	HR2125-12
	HR285-12
	HR2199-12
	HR2202-12
	HR2287-12
	HR2308-12
	HR2433-12
	HR2666-12
3.	HR15-13
	HR41-13
	HR129-13
	HR175-13
	HR235-13
	HR432-13
4.	HR2015-12

HR128-13
HR134-13
HR135-13
HR146-13
HR171-13
HR206-13
HR233-13
HR250-13
HR555-13
HR556-13
HR558-13
HR590-13
HR593-13
HR623-13
HR655-13

28. HR119-13
HR129-13
HR172-13
HR189-13
HR235-13
HR432-13
HR581-13

29. HR130-13
30. HR152-13
31. HR162-13
32. HR175-13
HR372-13
HR636-13

33. HR248-13
HR617-13
HR648-13

34. HR280-13
HR518-13

35. HR454-13
36. HR552-13
HR633-13

37. HR675-13
HR736-13

HR2146-12
HR2176-12

5. HR2058-12
HR2191-12
HR2249-12

6. HR186-13
HR372-13
HR636-13

7. HR248-13
HR617-13
HR648-13

8. HR110-15
HR239-13

9. HR119-13
HR152-13

10. HR128-13
HR233-13

11. HR675-13
HR736-13

12. HR7640-11
13. HR8934-11
14. HR7907-11
15. HR8203-11
16. HR9128-11
17. HR2051-12
18. HR2184-12
19. HR2328-12
20. HR2338-12
21. HR2375-12
22. HR2465-12
23. HR2488-12
24. HR2512-12
25. HR134-13
26. HR189-13
27. HR454-13
28. HR558-13
29. HR581-13
30. HR5-13

genotype ON1

group	strain name	group	strain name
1.	HR2095-12	1.	HR2095-12
2.	HR2117-12		HR194-13
3.	HR122-13		HR234-13
	HR598-13		HR84-14
4.	HR194-13		HR239-14
	HR234-13		HR242-14
	HR429-13		HR258-14
5.	HR354-13		HR266-14
6.	HR490-13		HR268-14
7.	HR620-13		HR478-14
8.	HR624-13		HR501-14
9.	HR641-13	2.	HR414-14
10.	HR718-13		HR457-14
11.	HR84-14		HR494-14
12.	HR102-14	3.	HR102-14
	HR307-14		HR307-14
13.	HR174-14	4.	HR174-14
	HR246-14		HR246-14
14.	HR196-14	5.	HR2117-12
15.	HR414-14	6.	HR354-13
	HR268-14	7.	HR429-13
	HR457-14	8.	HR490-13
	HR478-14	9.	HR598-13
	HR494-14	10.	HR620-13
	HR501-14	11.	HR624-13
16.	HR226-14	12.	HR641-13
17.	HR228-14	13.	HR718-13
	HR506-14	14.	HR196-14
18.	HR239-14	15.	HR226-14
	HR242-14	16.	HR228-14
19.	HR258-14	17.	HR491-14
20.	HR266-14	18.	HR122-13
21.	HR367-14	19.	HR367-14
22.	HR491-14	20.	HR506-14

genotype GA5

group	strain name	group	strain name
1.	HR358-14	1.	HR358-14
	HR423-14		HR423-14
	HR426-14		HR426-14

HRSV-B

sequences identical in HVR2		sequences identical in SHseg	
**genotype BA9**			
group	strain name	group	strain name
1.	HR6808-11	1.	HR586-13
2.	HR468-12		HR1765-13
	HR1019-12		HR1784-13
	HR2244-12		HR1895-13
	HR2264-12		HR1912-13
	HR2577-12		HR44-14
3.	HR1156-12		HR57-14
	HR1948-12		HR142-14
	HR2006-12		HR143-14
	HR2013-12		HR144-14
	HR2116-12		HR157-14
	HR2154-12		HR158-14
	HR2253-12		HR180-14
	HR2362-12		HR296-14
	HR2458-12		HR471-14
4.	HR1881-12		HR157-14
5.	HR1883-12		HR428-14
6.	HR2052-12		HR378-14
7.	HR2207-12	2.	HR468-12
8.	HR2248-12		HR1019-12
9.	HR2617-12		HR1156-12
	HR2637-12		HR2244-12
10.	HR3386-12		HR2264-12
	HR27-13		HR2577-12
	HR28-13		HR1156-12
	HR109-13		HR1948-12
	HR207-13		HR2006-12
	HR208-13		HR2013-12
	HR221-13		HR2253-12
	HR224-13		HR2362-12
	HR259-13		HR2458-12
11.	HR269-13		HR1883-12
	HR370-13		HR2052-12
	HR374-13		HR2207-12
12.	HR307-13		HR2248-12
13.	HR479-13	3.	HR2637-12
14.	HR501-13		HR27-13
15.	HR586-13		HR28-13
	HR1765-13		HR109-13
	HR1784-13		HR207-13
	HR1895-13		HR208-13
	HR1912-13		HR221-13
	HR44-14		HR224-13
	HR57-14		HR259-13
	HR122-14		HR370-13

	HR142-14
	HR143-14
	HR144-14
	HR158-14
	HR180-14
	HR207-14
	HR269-14
	HR296-14
	HR348-14
	HR402-14
	HR471-14
16.	HR804-13
17.	HR35-14
18.	HR40-14
	HR116-14
	HR189-14
	HR325-14
19.	HR63-14
	HR331-14
20.	HR74-14
21.	HR137-14
22.	HR157-14
	HR428-14
23.	HR200-14
24.	HR208-14
25.	HR233-14
26.	HR282-14
27.	HR287-14
28.	HR378-14
29.	HR492-14
30.	HR494-14
31.	HR530-14

genotype BA10

group	strain name
1.	HR2153-12

	HR501-13
4.	HR189-14
	HR331-14
	HR200-14
	HR282-14
	HR287-14
	HR492-14
5.	HR40-14
	HR325-14
	HR137-14
	HR233-14
6.	HR3386-12
	HR269-13
	HR374-13
7.	HR35-14
	HR208-14
8.	HR74-14
	HR122-14
9.	HR269-14
	HR402-14
10.	HR6808-11
11.	HR1881-12
12.	HR2154-12
13.	HR2617-12
14.	HR307-13
15.	HR479-13
16.	HR804-13
17.	HR63-14
18.	HR116-14
19.	HR207-14
20.	HR494-14
21.	HR530-14

genotype BA10

group	strain name
1.	HR2153-12
